# Effect of C-type lectin 16 on dengue virus infection in *Aedes aegypti* salivary glands

**DOI:** 10.1093/pnasnexus/pgae188

**Published:** 2024-05-16

**Authors:** Ya-Chen Chang, Wei-Liang Liu, Pai-Hsiang Fang, Jian-Chiuan Li, Kun-Lin Liu, Jau-Ling Huang, Hsin-Wei Chen, Chih-Fei Kao, Chun-Hong Chen

**Affiliations:** National Mosquito-Borne Diseases Control Research Center, National Health Research Institutes, Miaoli 35053, Taiwan; National Institute of Infectious Diseases and Vaccinology, National Health Research Institutes, Miaoli 35053, Taiwan; National Mosquito-Borne Diseases Control Research Center, National Health Research Institutes, Miaoli 35053, Taiwan; National Institute of Infectious Diseases and Vaccinology, National Health Research Institutes, Miaoli 35053, Taiwan; National Institute of Infectious Diseases and Vaccinology, National Health Research Institutes, Miaoli 35053, Taiwan; National Mosquito-Borne Diseases Control Research Center, National Health Research Institutes, Miaoli 35053, Taiwan; Department of Bioscience Technology, Chang Jung Christian University, Tainan 711301, Taiwan; National Institute of Infectious Diseases and Vaccinology, National Health Research Institutes, Miaoli 35053, Taiwan; Department of Biological Science and Technology, National Yang Ming Chiao Tung University, Hsinchu 300093, Taiwan; National Mosquito-Borne Diseases Control Research Center, National Health Research Institutes, Miaoli 35053, Taiwan; National Institute of Infectious Diseases and Vaccinology, National Health Research Institutes, Miaoli 35053, Taiwan

**Keywords:** CRISPR/Cas9, *Aedes aegypti*, C-type lectins, CTL16, transmission

## Abstract

C-type lectins (CTLs) are a family of carbohydrate-binding proteins and an important component of mosquito saliva. Although CTLs play key roles in immune activation and viral pathogenesis, little is known about their role in regulating dengue virus (DENV) infection and transmission. In this study, we established a homozygous *CTL16* knockout *Aedes aegypti* mutant line using CRISPR/Cas9 to study the interaction between *CTL16* and viruses in mosquito vectors. Furthermore, mouse experiments were conducted to confirm the transmission of DENV by *CTL16^−/−^ A. aegypti* mutants. We found that *CTL16* was mainly expressed in the medial lobe of the salivary glands (SGs) in female *A. aegypti*. *CTL16* knockout increased DENV replication and accumulation in the SGs of female *A. aegypti*, suggesting that *CTL16* plays an important role in DENV transmission. We also found a reduced expression of immunodeficiency and Janus kinase/signal transducer and activator of transcription pathway components correlated with increased DENV viral titer, infection rate, and transmission efficiency in the *CTL16* mutant strain. The findings of this study provide insights not only for guiding future investigations on the influence of CTLs on immune responses in mosquitoes but also for developing novel mutants that can be used as vector control tools.

**Significance Statement**

The C-type lectin (CTL) superfamily comprises various proteins that can bind to viral particles or infected cells to initiate innate immune responses in mosquitoes, limiting arbovirus replication and dissemination. Modulation of CTL expression may alter *Aedes aegypti* midgut microbiota, which plays an important role in viral infectivity. Understanding the interactions between CTLs and pathogens may facilitate the development of novel approaches for controlling mosquito-borne diseases and reducing their transmission.

## Introduction

Dengue fever is mainly transmitted by *Aedes aegypti* mosquitoes, and their salivary glands (SGs) are an important route for virus transmission. The mosquito SG comprises three discernible regions: the proximal lateral lobe (PL), distal lateral lobe (DL), and medial lobe (ML) (1). Mosquito SGs have a paired architecture and are much larger in females than in males. The three lobes of the male SG are similar and involved in carbohydrate absorption and digestion. In female mosquitoes, each region of the SG plays distinct roles in their sugar- and blood-feeding behaviors. The proximal lateral lobe participates in the synthesis and secretion of blood meal digestion–promoting enzymes, such as amylase, α-1,4-glucosidase, and glycosidases, which help in nutrient acquisition. The DL is primarily involved in the secretion of antihemostatic compounds, such as apyrases and serine proteases, which prevent host blood clotting to allow efficient blood meal acquisition by mosquitoes. Meanwhile, the ML is associated with the secretion of saliva, which assists the probing and feeding processes in mosquitoes (2, 3). Further enhancing the mosquito's feeding success, the expression of C-type lectins (CTLs) in its SGs has garnered significant attention.

CTLs are a family of carbohydrate-binding proteins that play important roles in immune responses and viral pathogenesis in various organisms, including mosquitoes such as *Aedes* species (4, 5). These lectins recognize and bind to specific carbohydrate structures on pathogens, triggering immune responses to eliminate or control the infection. CTLs are key components of mosquito saliva (6, 7), which is injected into the host during blood sucking. CTLs in mosquito saliva influence the host immune response (8) and, thus, pathogen transmission (9, 10). However, the function and mechanism of CTLs in mosquito saliva remain unclear.

*Aedes aegypti* mosquitoes, carriers of the dengue virus (DENV), possess over 52 types of CTL proteins. Some of these, such as mosGCTL-1, mosGCTL-3, and mosGCTL-7, are critical for virus infection processes (11, 12). These CTLs facilitate viral attachment and entry: mosGCTL-1 interacts with a mosquito protein similar to human CD45, aiding West Nile virus infection (13); mosGCTL-3 binds to the DENV, enhancing infection and affecting mosquito health when absent (5, 14); mosGCTL-7 facilitates Japanese encephalitis virus infection through cell surface interaction (15). Comparable mechanisms in humans involve CTLs like CLEC5A, which recognize viruses and potentially lead to severe dengue fever through inflammation activation (16).

Mosquitoes have four major pathways of innate immune response against viral infections: the Toll, immunodeficiency (IMD), Janus kinase/signal transducer and activator of transcription (JAK/STAT), RNA interference (RNAi) pathways, and c-Jun N-terminal kinase pathways (17–19). CTLs are important pattern recognition receptors (PRRs) in mosquitoes that can activate different immune pathways to elicit various responses (20, 21). In *Aedes* mosquitoes, CTLs act as immune antagonists and can help the gut microbiome evade the bactericidal activities of antimicrobial peptides (AMPs), thus protecting the microbial flora (22, 23). Although JAK/STAT- and Toll-regulated AMPs are significantly up-regulated in DENV-infected mosquitoes, DENV-infected cells down-regulate IMD-regulated AMPs (24–26). The interactions between the different signaling pathways are highly complex and interrelated. However, the roles of different CTLs in modulating these pathways remain relatively unexplored. Therefore, further research on the roles of CTLs in the mosquito immune system will be worthwhile.

Previous studies using RNA in situ hybridization have provided compelling evidence for the presence of RNA of *CTL16* (AAEL000533) in mosquito SGs (27, 28). Proteins targeting CTL domain–containing proteins are classified into three classes, namely CTLD-S, CTLD-SP, CTLD-E and CTLD-X, which increases the possibility of protecting the gut microbiome and facilitating flavivirus infection (11, 12, 23). However, *CTL16* contains the CTLD-S domain. A recent study showed up-regulation and down-regulation of *CTL16* in the SGs of Zika virus (ZIKV)-infected and DENV-2-infected mosquitoes, respectively (29). A phylogenetic analysis revealed 44% sequence similarity between the amino acid sequences of *CTL16* and mosGCTL-3 (30). Since *CTL16* is highly homologous to mosGCTL-3, a potential DENV PRR, it may have similar functions in the immune response against pathogens in mosquitoes (30).

In this study, we generated *CTL16* knockout *A. aegypti* mutants using CRISPR/Cas9 to study the effects of *CTL16* on DENV replication in mosquito SGs and the associated signaling pathways and confirmed our findings by animal transmission studies. Investigating other CTL-knockout mosquito lines in the future will provide further insights into the function and mechanism of this protein family in relation to DENV and other arboviruses.

## Results

### *CTL16* localization in SGs

**Fig. 1.**
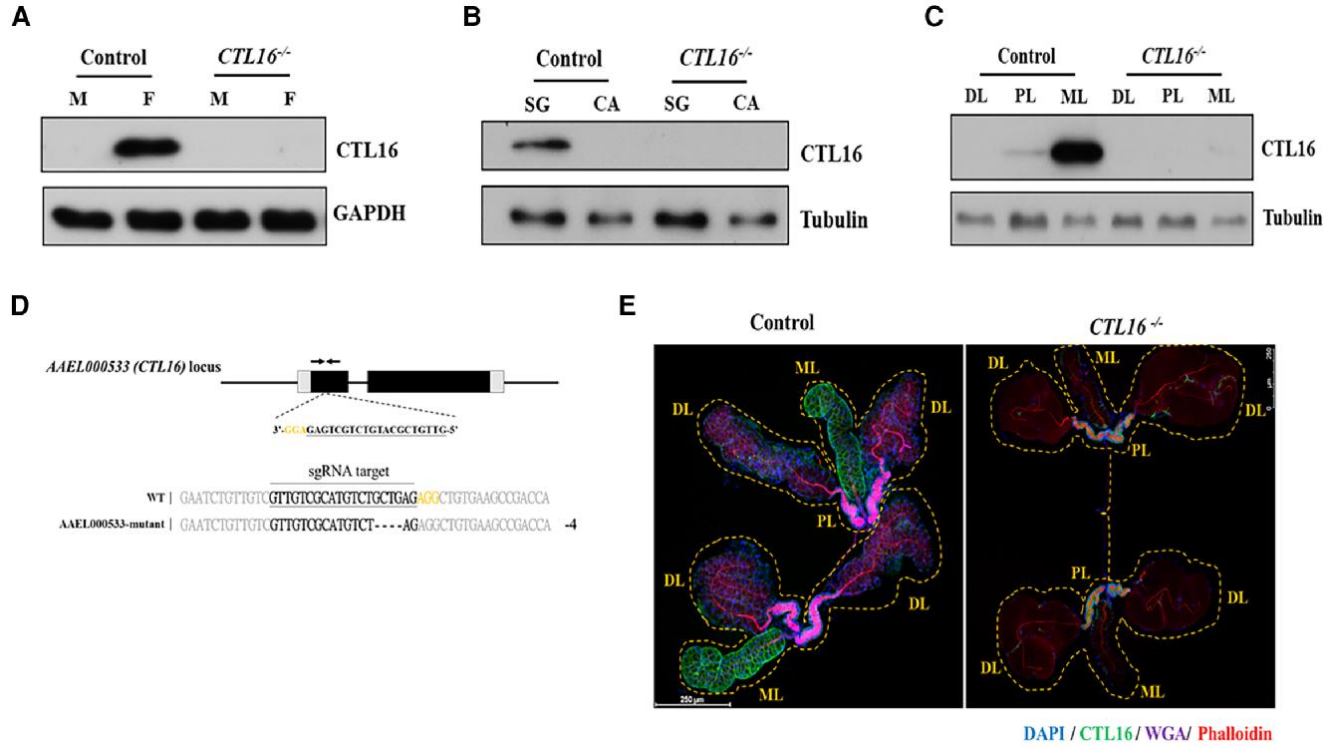
*CTL16* expression in *A. aegypti* SG. A) Western blotting for *CTL16* in samples from male (M) and female (F) *CTL16* knockout mutant and wild-type mosquitoes using self-made *CTL16* antibody and antiglyceraldehyde 3-phosphate dehydrogenase as the internal standard. B) *CTL16* expression in SGs and carcasses of female mosquitoes. In (A) and (B), we pooled SG from five mosquitoes for each test and conducted three replicate tests for each experiment. C) *CTL16-*specific localization in SGs. We analyzed three regions of the *A. aegypti* SG (PL, proximal lateral lobe; DL, distal lateral lobe; ML, medial lobe) by western blotting. Each test involved pooling individual regions of SG lobes from 15 mosquitoes, with each experiment repeated 3 times. The generation of *CTL16* knockout *A. aegypti* by CRISPR/Cas9. D) A schematic diagram of *A. aegypti CTL16* locus showing sgRNA target sites (black bottom line). Alignment of the *CTL16* intron 2 border and the mutated DNA sequence shows a 4 bp deletion (dashed black line). Deletions appear in frameshift mutations and a premature stopping of translation upon encountering a stop codon. E) Analyzing the localization of *CTL16* through immunofluorescence staining. SGs (*n* = 20) from 5-day-old female mosquitoes with *CTL16* knockout mutation were collected and dissected. A staining assay was conducted as described in the Materials and Methods section. Scale bar = 250 µm. SG, salivary gland; CA, carcass.

*CTL16* is an SG protein expressed in adult female *A. aegypti*. However, the exact localization of *CTL16* in *A. aegypti* SGs remains unclear. To ascertain the exact localization of the *CTL16* protein within the SGs, a western blot analysis of samples from both male and female specimens using anti-*CTL16* antibodies was performed, which revealed that *CTL16* was exclusively expressed in female *A. aegypti* (Fig. 1A). Furthermore, *CTL16* expression was exclusively detected within the SGs, as confirmed through dissection (Fig. 1B). Next, to determine the precise localization of *CTL16* within the SGs, we conducted an analysis of three distinct regions: the PL, the DL, and the ML. Our study pinpoints the *CTL16* protein’s specific location within the ML of the SG, elucidating its spatial distribution within this intricate SG structure (Fig. 1C).

### Generation of *CTL16* knockout mutant *A. aegypti*

In existing studies, there are indications that CTLs are involved in arbovirus infections (20). Therefore, we aim to further investigate this aspect by generating *CTL16* knockout mutant strains in mosquitoes and studying their related functions. A *CTL16* knockout (*CTL16*^−/−^) mutant *A. aegypti* line was created using CRISPR/Cas9 technology (AAEL006511-Cas9) with a single-guide RNA (sgRNA) plasmid, which was microinjected into embryos to induce a targeted indel (31). The sgRNA targeted exon 1 near the 5′-untranslated region of *CTL16* to eliminate the production of a partially functional protein. PCR and sequencing confirmed homozygous *CTL16* knockout in the resulting mutant *A. aegypti* line and unveiled a four-base deletion leading to a stop codon and subsequent protein non-expression (Fig. 1D). A western blot analysis corroborated *CTL16* non-expression in the mutant line (Fig. 1A–C). An immunofluorescence assay with confocal microscopy showed distinct *CTL16* signals in the ML of SGs in wild-type female *A. aegypti* but not in *CTL16*^−/−^ mutant female *A. aegypti* (Fig. 1E). However, the anatomical integrity of the SGs in mutants remained unaltered (Fig. S1). Cumulatively, these findings corroborate the specific expression of *CTL16* within the SGs of female mosquitoes.

### Loss of *CTL16* increases viral titers and E-protein expression

**Fig. 2.**
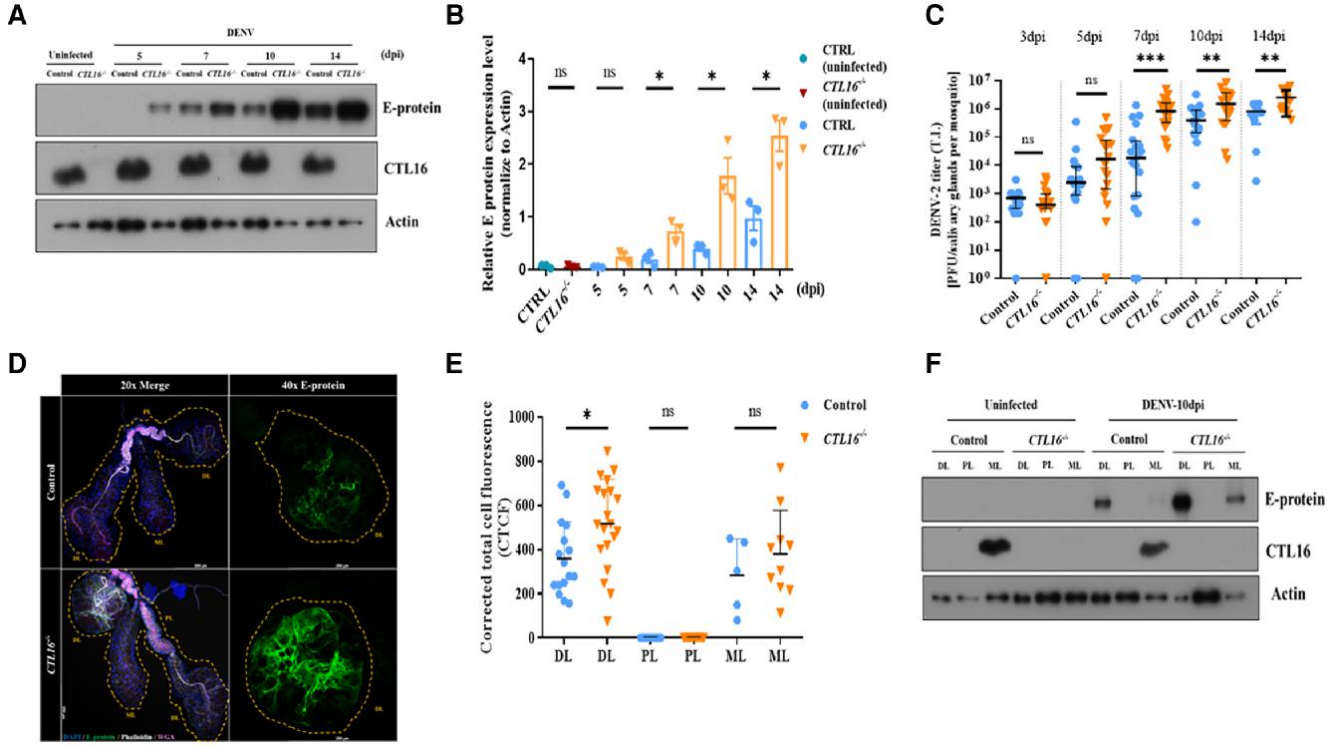
*CTL16* ^−/−^ transgenic mutant mosquitoes exhibit increased E-protein expression and viral titers. A) A western blot was used to determine the expression of the E protein in control and *CTL16^−/−^* mosquitoes after thoracic injection of DENV-2. SGs pooled from five mosquitoes were collected for each experiment and subjected to three replicate tests. B) Quantification of E-protein levels in (A) was normalized to β-actin levels. Each data point represents the average titer of five mosquitoes. The data are presented as the mean ± SD of E-protein expression levels. C) DENV challenge in the control and *CTL16^−/−^* mutant mosquitoes. Twenty SGs were collected at each time point for subsequent plaque formation assays. D) Localization of DENV E-protein expression in *CTL16^−/−^* mosquitoes. At 10 dpi, SGs (*n* = 20) were collected and subjected to immunofluorescence staining. Scale bar = 250 µm. E) A quantitative analysis of (D). Confocal images were processed using ImageJ and analyzed following the formula in the Materials and Methods section. F) At 10 dpi, the protein expression levels of DENV-2 in three regions of the SGs in control and *CTL16^−/−^* mutant mosquitoes were tested by western blotting. Each test involved pooling individual regions of SG lobes from 15 mosquitoes, with each experiment repeated three times. ns, no significant difference; DL, distal lateral lobes; PL, proximal lateral lobes; ML, medial lobes. The asterisks represent significant differences between the two mosquito lines: *P < 0.05, **P < 0.01, ***P < 0.001.

The CTL superfamily contains multiple proteins that play important roles in combating viral infections (13, 14). To investigate the role of *CTL16^−/−^* mutant lines in modulating DENV titers, we examined the association between *CTL16* and DENV proteins. Western blotting showed increased E-protein expression after DENV-2 administration via thoracic injection in the *CTL16^−/−^* mutant line compared with that in wild-type *A. aegypti* (Fig. 2A and B). The E-protein level in *CTL16^−/−^* mutants was up to 3-fold higher than in wild-type *A. aegypti* at 7 days’ post infection (dpi), and the level remained higher at 10 and 14 dpi. A plaque formation assay revealed significantly higher median virus titers in the SGs of *CTL16^−/−^* mutants than in those of wild-type individuals at 7, 10, and 14 dpi (Fig. 2C).

To visualize and quantify the accumulation of DENV in the SGs, an immunofluorescence assay was conducted. Our studies showed E-protein accumulation in both the DL and the ML of the SGs at 10 dpi (Fig. 2D and E). A corrected total cell fluorescence analysis confirmed a significantly higher E-protein expression in the DL of *CTL16^−/−^* mutants compared with that of wild-type individuals. A western blot analysis of the SGs after 10 dpi further supported these findings (Fig. 2F). These results suggest that the absence of *CTL16* may facilitate DENV-2 expression in the DL of the SGs and its initial replication and accumulation in the ML, potentially affecting the overall virus expression in the SGs.

### *CTL16* ^−/−^ mosquitoes increase the DENV infection rate in AGB6 mice

**Fig. 3.**
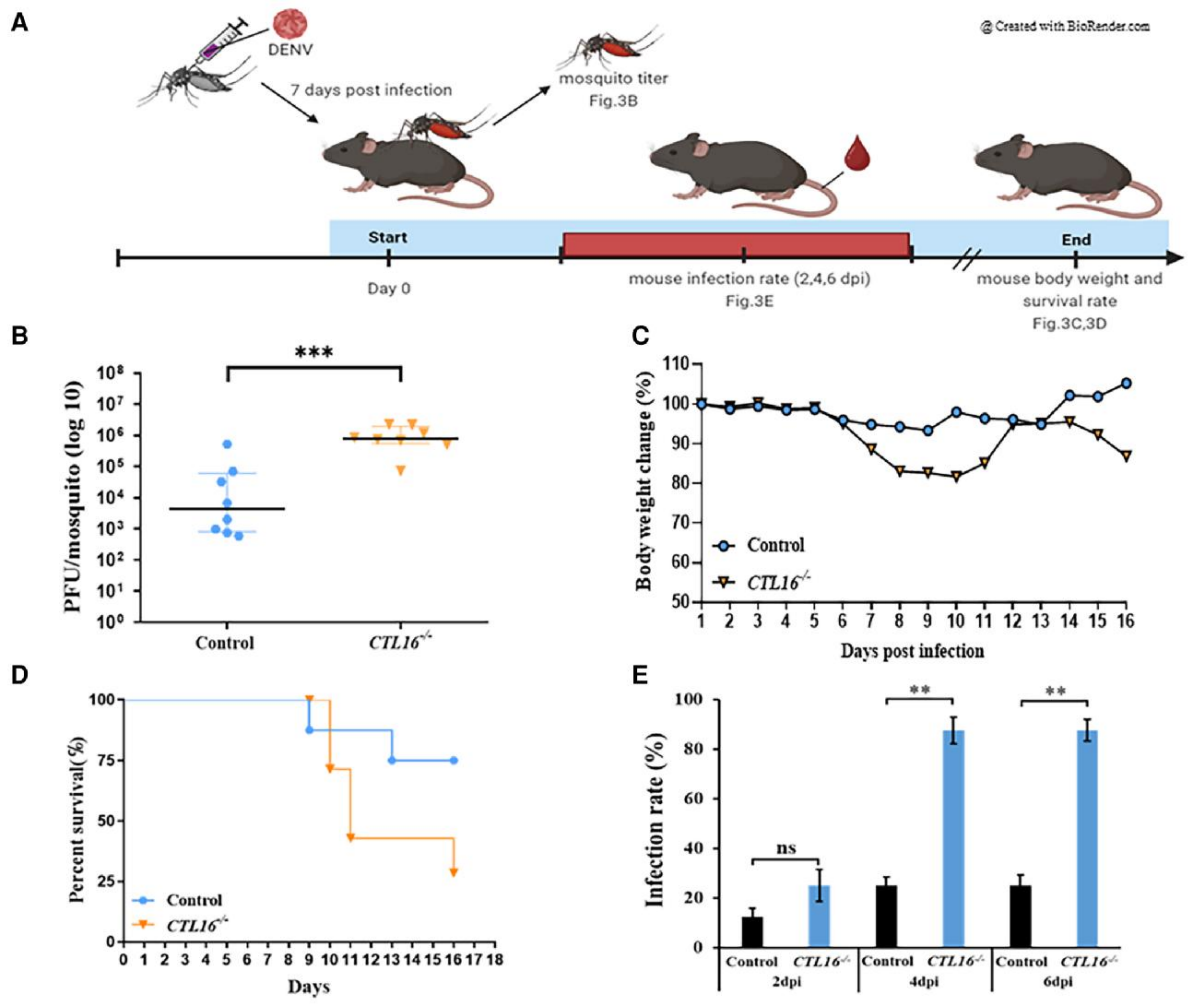
*CTL16* ^−/−^ mosquitoes increase the susceptibility of AGB6 mice to DENV. A) A flow chart of the experiment for mosquito-to-mouse DENV-2 transmission. B) Virus testing on mosquitoes that bite mice. Seven days after thoracic injection with DENV-2, control, and *CTL16^−/−^* mutant mosquitoes fed on mice. In each experiment, four mosquitoes were collected, and two replicate tests were conducted. Each dot represents the viral titer test for each mosquito. C) Body weight and D) survival in AGB6 mice (*n* = 8) bitten by DENV-2-infected mosquitoes. E) IRs of the mice were defined as the percentage of virus-positive mouse samples out of the total mice tested (*n* = 8). A t test was performed in the analysis of the difference in mouse IRs, and in the mosquito transmission efficiency assay. The error bars correspond to the 95% CIs. The asterisks represent significant differences between the genotypes: **P < 0.01, ***P < 0.001.

Mature DENVs can replicate in the mosquito's SG and then be transmitted to humans through the bites of these infected mosquitoes (10, 32). Our studies have also shown that *CTL16* is expressed in the SG and affects DENV performance (Figs. 1 and 2). To further understand whether *CTL16* can enhance the mosquito's ability to transmit DENV, we injected DENV-2 into the mosquito's thorax and allowed them to feed on AGB6 mice (deficient in expression of interferon receptor types I and II on a C57BL/6 genetic background) (33) at 7 dpi. The mosquitoes were later harvested to determine viral titers. We monitored mice body weight and survival daily and collected mouse sera at 2, 4, and 6 dpi to determine viral titers using a plaque-forming assay (Fig. 3A). Our findings indicate that the SG virus titer in *CTL16^−/−^* mutant mosquitoes was higher than in wild-type individuals (Fig. 3B). Mice bitten by *CTL16* mutant mosquitoes infected with DENV-2 showed a nearly 20% decrease in body weight from day 5 to day 10, with no significant change observed in the control group (Fig. 3C). Additionally, the mortality rate of mice bitten by *CTL16* mutant mosquitoes carrying DENV-2 increased from 25% on day 10 post bite to 50% on day 11 and reached 75% on day 16 post bite. In contrast, the control group had a mortality rate of 37.5% on day 16 post bite (Fig. 3D). Experimental data on mosquito bites showed that mice bitten by *CTL16^−/−^* mutants exhibited lower body weight and survival rates than those bitten by wild-type mosquitoes. Additionally, we analyzed the virus titer in the mice and found no significant difference compared with the control group after being bitten by mosquitoes of the *CTL16^−/−^* mutant line. However, there was a significant difference in the infection rate (IR), defined as the number of virus-positive mice among the total mice tested (Fig. 3E). The *CTL16^−/−^* mutant line exhibited a substantially increased IR relative to the control line at 4 dpi (25.0 ± 1.7 vs. 87.5 ± 5.8%, *P* < 0.01), 6 dpi (25.0 ± 2.1 vs. 87.5 ± 3.9%, *P* < 0.01), whereas no effect was noted at 2 dpi (12.5 ± 2.1 vs. 25.0 ± 5.4%, *P* = 0.054). In conclusion, these findings demonstrate that *CTL16* may influence DENV transmission by mosquitoes.

### Virus transmission efficiency of *CTL16*^−/−^ mosquitoes

In animal experiments, it was observed that mosquitoes carrying *CTL16^−/−^* mutant lines were capable of transmitting the virus to mice. To assess the transmissibility of these *CTL16^−/−^* mutant mosquitoes, we collected the SGs and saliva from mosquitoes 7 days after infection with DENV-2 to test viral titers and transmission efficiency (Fig. S2). In the SGs of the *CTL16^−/−^* mutant lines, DENV-2 viral titers were higher than those in the control group at 7 dpi (Fig. S2A). After confirming potential enhancements in the SGs, we proceeded to test the viral titer in saliva. In the saliva samples, we found that both the *CTL16^−/−^* mutant line and the control group had detectable viral titers of DENV-2, which increased at 7 dpi (Fig. S2B). We also observed that the *CTL16^−/−^* lines exhibited significantly higher transmission efficiency compared with the control mosquitoes (Fig. S2C). These experimental results collectively indicate that *CTL16* significantly affects the transmission efficiency of DENV-2.

### *CTL16* is capable of binding to DENV-2

**Fig. 4.**
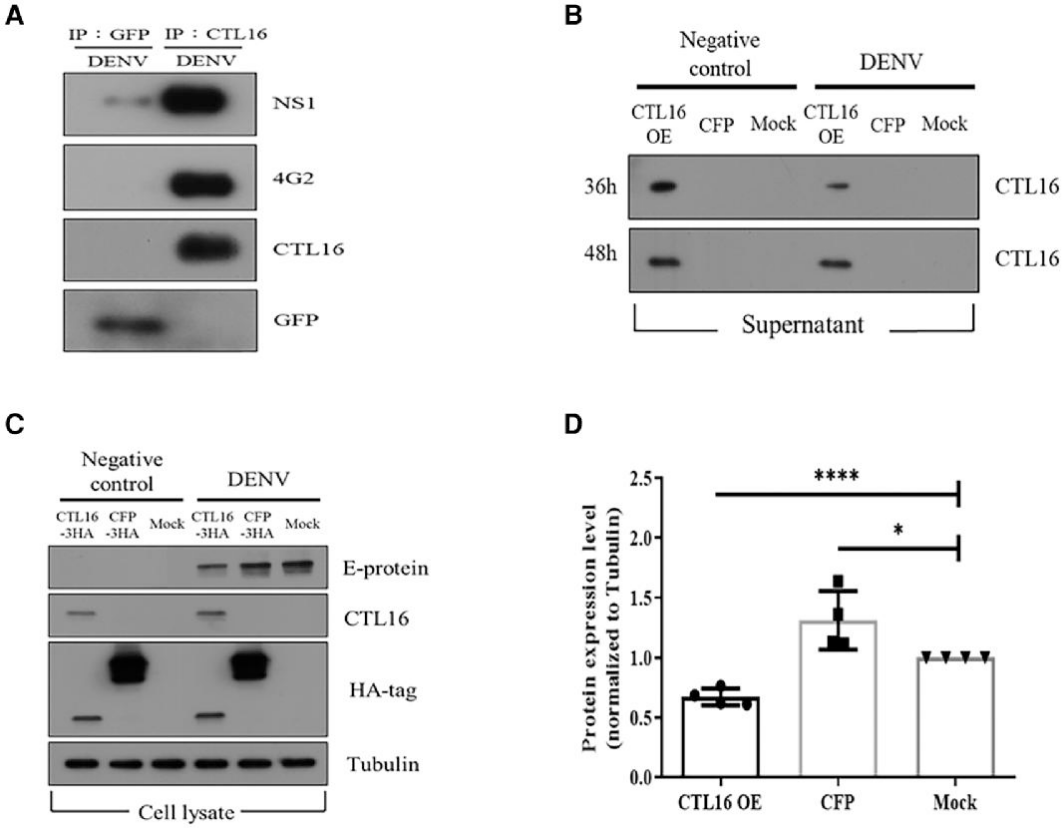
*CTL16*: binding, secretion, and suppression of DENV replication. A) *CTL16* and DENV NS-1 and E protein interaction: this demonstrates *CTL16* binding to dengue NS-1 and E proteins using immunoprecipitation assays with anti-GFP and anti-*CTL16*, followed by western blots with specific antibodies. This confirms the formation of the *CTL16*–virus complex, with GFP serving as a negative control. B) Posttransfection *CTL-16* secretion in C6/36 cells: observes *CTL16* expression and secretion in C6/36 cells transfected with *CTL16* and CFP, with and without DENV-2 infection. Anti-*CTL16* antibodies in western blots assess *CTL16* in supernatants 36 and 48 h posttransfection. C) *CTL16's* impact on DENV-2 in C6/36 cells: evaluates *CTL16*’s potential to inhibit DENV-2, analyzing cell lysates from C6/36 cells, with or without DENV-2 infection, focusing on E-protein levels as a measure of inhibition. D) E-protein reduction by *CTL16*: quantifies dengue E-protein reduction in *CTL16*-expressing C6/36 cells, normalized to α-tubulin, showing a significant E-protein decrease compared with controls (*n* = 3). The results are mean ± SD. Successful *CTL16* and CFP transfections were confirmed via an HA antibody, using tubulin as an internal control. *CTL16*-3HA: *p*AC5.1_*CTL16*-3HA vector; CFP-3HA: *p*AC5.1_CFP-3HA vector; Mock: *p*AC5.1 vector. The asterisks indicate significant differences (*P < 0.05, ****P < 0.0001; unpaired t test), highlighting CTL16’s effectiveness in lowering dengue E-protein levels.

Figure 2 indicates that the SG of *CTL16^−/−^* mosquitoes can accumulate a large amount of DENV. We hypothesize that *CTL16* may interact with the DENV. The immunoprecipitation assay showed that the NS-1 and E proteins of DENV-2 can interact with *CTL16* (Fig. 4A). Next, we simultaneously collected both supernatants and cells in the cell culture system to investigate whether *CTL16* can be released from cells and its potential inhibitory effect on DENV within the cells. We validated this using *Aedes albopictus* C6/36 cells (34) transfected with either the CFP vector or the *CTL16* plasmid and challenged with or without DENV-2 (control). Experiments involving the collection of supernatants at various time intervals have unveiled the potential release of *CTL16* into the extracellular culture medium (Fig. 4B), suggesting *CTL16's* role as a secreted protein. This finding indicates its possible involvement in intercellular processes. Furthermore, intracellular experimental observations revealed that the overexpression of *CTL16* significantly diminished the levels of DENV-2 E protein compared with the control group (Fig. 4C), suggesting its potential to suppress DENV replication intracellularly. Figure 4D offers a quantitative depiction corresponding to the results presented in Fig. 4C. Collectively, these findings suggest that *CTL16* may interact with DENV proteins, thereby regulating the levels of DENV in mosquitoes.

### Innate immune pathways are down-regulated in *CTL16*^−/−^ mosquitoes

CTLs act as essential components of many innate immune systems and are recognized as PRRs for various viruses, including DENV. They activate specific pathways to regulate virus expression (23, 24, 35). Figures 2 and S3 reveal up-regulated DENV replication and production in *CTL16^−/−^* mosquitoes, suggesting that *CTL16* may have a regulatory role in mosquito immunity. We, therefore, studied whether *CTL16^−/−^* interacts with the innate immune pathway and affects DENV infection. Quantitative real-time PCR with RPS7 as a reference showed that the JAK/STAT pathway components Dome and Hop were down-regulated in the whole SGs of *CTL16^−/−^* mutant mosquitoes infected with DENV-2 via blood meals at 1–7 dpi. The IMD pathway component Fas-associated death domain (FADD) was also down-regulated in *CTL16^−/−^* mutant mosquitoes at 5 and 7 dpi (Fig. S4A). Furthermore, we also conducted corresponding immune analysis on the three lobes of SG (Fig. S4B–D). In the DL, Dome/Hop and FADD showed a decrease in the early stages (1–3 dpi) compared with the control. In the ML, Dome was affected on 1 dpi, while FADD was affected on 5 dpi. The immune response in the proximal lateral lobe appeared to remain unchanged. However, other components of the Toll and RNAi pathways were not altered in *CTL16^−/−^* mutants at any point in time. These results indicate that the expression of IMD and JAK/STAT pathway-related components is reduced in *CTL16^−/−^* mutants. Moreover, the expression levels of these components were apparently influenced by the increases in other factors, such as DENV titers and transmission rates.

## Discussion

Dengue fever is one of the most prevalent arthropod-borne viral diseases, affecting ∼50 million people worldwide per year. Over 2.5 billion people are currently at risk of DENV infection. DENV is transmitted to humans through a female *A. aegypti* or *A. albopictus* bite. Mosquitoes contract the virus when feeding on the blood of an infected person. The virus then infects the midgut of the mosquito and starts to disseminate via hemolymph to infect other tissues. Finally, the virus reaches the SGs, where it replicates. The virus is then transmitted to the next host via mosquito saliva within 10–14 days (34–36). Preventing virus replication in mosquito tissues represents an important strategy to reduce DENV transmission to humans. Previous studies have demonstrated the significance of both mosquito and human CTLs in the efficacy of anti-DENV and anti-ZIKV approaches (5, 14, 37). In the present study, *CTL16* knockout in mosquitoes using CRISPR/Cas9 impacted DENV replication and accumulation in mosquito SGs, thereby influencing virus transmission. Thus, the introduction of anti-DENV genes into mosquito populations may represent another approach for controlling DENV.

In this study, immunostaining for DENV E protein in SGs revealed that the DL is usually the first site to be infected and that the virus spreads to the MLs from there (Figs. 2F and S3). These findings are consistent with those of Salazar et al. (35) and Raquin and Lambrechts (32). The fluorescence intensity increased significantly in the DL of *CTL16^−/−^* mosquitoes at 10 dpi. At 14 dpi, significant differences were observed in fluorescence intensity between the DL and ML regions of the SG, with minor changes detectable in the proximal lobe. This suggests that *CTL16* influences the early stages of DENV infection in the SGs.

Although *CTL16* is not present in the DL, it has been identified as a secreted product from C6/36 cells. These cell-based experiments have established *CTL16's* function as a secretory protein, as illustrated in Fig. 4B. Additionally, immunoprecipitation experiments, described in Fig. 4A, have shown recombinant *CTL16's* capability to bind to the DENV. Given CTL proteins’ potential in initiating antiviral defensive mechanisms by acting as PRRs and triggering immune responses, *CTL16's* interaction with the DENV holds considerable significance. It is suggested that *CTL16*, upon being secreted by the ML, might directly or indirectly interact with DENV within the DL, thus stimulating immune reactions that may lower the virus's proliferation or IRs. Furthermore, *CTL16* dispersing into the hemocoel could potentially engage with DENV in various tissues, promoting the spread of immune signals and leading to antiviral effects. This concept finds support from our observations in *CTL16^−/−^* mutant lines, which showed reduced immune responses and, as a result, elevated viral loads (Figs. 2 and S4). Therefore, *CTL16* may be considered a PRR for various viruses, regulating virus expression by initiating specific immune pathways or other antiviral pathways. However, the detailed regulatory mechanisms are not clear and worth further investigation in future in vivo experiments, which could provide more detailed insights into the mechanisms of CTL proteins’ function.

Hosts, including insects, have complex immune systems to defend against pathogen invasion. Insects particularly rely on innate immunity and cytokine-like responses for defense against viral infection (38, 39). Previous studies reported that CTLs can induce immune responses against DENV infection via the IMD, Toll, JAK/STAT, and RNAi pathways in mosquitoes. Knockdown of mos*GCTL-3* has been reported as up-regulate JAK/STAT signaling and increase the production of certain AMPs in mosquitoes, reducing the prevalence of DENV-2 infection (14). Human CLEC18A expression in mosquitoes up-regulated many Toll, JAK/STAT, and RNAi pathway components, reducing DENV IR in transgenic lines (40). Interestingly, loss-of-function mutation for *CTL16* in mosquitoes inhibited downstream expression of the transcription factors FADD, Dome, and Hop in the IMD and JAK/STAT pathways (Fig. S4), thereby affecting DENV replication. The roles of different CTLs in regulating these pathways remain unclear (14). Understanding the influence of CTLs on immune responses in mosquitoes may help develop new transgenic lines as tools for vector control.

The previous publication discussed the roles of various immune signaling pathways and their downstream factors in limiting the spread of pathogens following infection (17). When the downstream signaling factors Dome/Hop of the JAK/STAT pathway form a complex, they recruit STAT to induce STAT phosphorylation and dimerization, ultimately activating the expression of immune genes and reducing viral infection. Therefore, silencing Dome or Hop makes mosquitoes more susceptible to DENV infection (41). This is similar to our results. In *CTL16^−/−^* mutant mosquitoes, the expression levels of Hop and Dome decrease, potentially hindering the effective immune function of the JAK/STAT pathway against viral infection. Furthermore, we also found that transgenic mosquitoes with *CTL16* loss of function suppress the transcription factor FADD in the IMD pathway. Studies have reported that FADD is a death domain protein in the *A. aegypti* and may serve as an intracellular adaptor essential for FADD-mediated cell apoptosis signal transduction (42, 43). This pathway may lead to the down-regulation of apoptosis markers, potentially increasing DENV infection. This observation may differ from the previously known IMD signaling pathway, which induces AMP expression for antiviral activity (44). This indicates that another innate immune defense mechanism is also affected. Overall, our results suggest that *CTL16* regulates mosquito immune responses, thereby controlling the replication of DENV.

The carbohydrate-binding sites of CTLs can bind to membrane proteins on viruses of the genus *Flavivirus* or increase viral attachment to modulate immune responses (13). Previous studies have reported the activation of Toll, IMD, and JAK/STAT pathways to stimulate the expression of AMPs that combat viral infection via different mechanisms (45, 46). Our results concur with these reports, as shown in Figs. 4 and S4. *CTL16* can bind to the NS-1 and E proteins of DENV-2, which may promote the expression of AMPs that can be induced by immune responses to achieve antiviral activity.

In this study, the mutant mosquito line exhibited a modest reduction in the mRNA expression levels of Dome, FADD, and HOP, as depicted in Fig. S4. Despite this, there was approximately a 10-fold increase in viral titers, suggesting the involvement of additional antiviral mechanisms not accounted for by the expression of these immune responses. Research indicates that CTLs can recognize and bind to HIV virus particles, potentially interfering with the virus's ability to neutralize host defenses (47). Similarly, it has been shown that CTL16 can bind to the DENV through its envelope (E) protein (Fig. 4A), implying that CTL16 might also possess the capability to bind to and neutralize viral particles. Recent studies have reported that DENV and ZIKV can infect phagocytic hemocytes in *A. aegypti* (48), facilitating the viruses’ dissemination over long distances. This study raises the possibility that CTL16, which can be secreted from cells, might block these infections in hemocytes or other tissues, a hypothesis that warrants further investigation. CTLs might also impact virus replication directly, possibly by inhibiting the activity of viral RNA polymerase or by blocking other essential replication factors (49), thereby impairing the virus's ability to replicate and spread within host cells. These preliminary findings highlight several potential mechanisms through which CTL16 could exert antiviral effects. However, more research is necessary to definitively ascertain CTL16's roles and to explore how these mechanisms can be processed, which is crucial for advancing our approaches to disease prevention.

According to previous studies, mosGCTLs not only affect immune pathways but also the mosquito microbiota, and thus, viral titers and development (14, 50). For example, increased secretion by *Serratia marcescens* can compromise the mucin barrier of the midgut epithelium and promote DENV infection, whereas treatment with antibiotics reduces DENV susceptibility in mosquitoes. The presence of *S. marcescens* is crucial for DENV infection. Moreover, *S. marcescens* can cause host diseases and affect the growth, survival, and development of mosquito larvae (51). Dysbiosis of the midgut microbiota in mosquitoes is associated with changes in the Toll pathway and immune-related expression of several AMPs (52). Therefore, the CTLs in mosquito have a significant and non-negligible impact on antiviral element. They can influence the balance of bacterial populations in the mosquito gut, thereby affecting DENV infectivity. In future studies, next-generation sequencing may provide further insights into the differences in the gut microbiomes of *CTL16*-expressing and *CTL16*^−/−^ mosquitoes.

In summary, we established a mutant *A. aegypti* line using CRISPR/Cas9 and investigated the relationship between *CTL16* and arbovirus infection. Our results demonstrate that DENV infectivity and transmission efficiency were enhanced in *CTL16*^−/−^ mosquitoes. These findings are consistent with the results of the animal experiments. Furthermore, *CTL16* exhibited the ability to bind to DENV viral proteins and particles, potentially enhancing the IMD and JAK/STAT innate immune pathways and up-regulating the expression of AMPs, which are important for preventing DENV infection. Since there is a lack of effective methods for dengue fever control, new vector control methods are urgently needed to combat the spread of DENV. Transgenic mosquitoes that can suppress DENV replication hold great promise in this regard.

## Materials and methods

### Ethics statement

Experimental animals were provided by Dr Guann-Yi Yu's lab at the National Health Research Institute (approval ID, #107054). All experiments and mouse rearing were performed according to the guidelines set by the Institutional Animal Care and Use Committee. All researchers involved in this study had completed the Animal Care Training course provided by the National Health Research Animal Center before beginning the experiments.

### Mosquito rearing

All experiments used Liverpool (*A. aegypti* strain) as the control strain and mutants derived from this strain. Mosquito larvae were reared at 28°C and fed a mixture of yeast powder (Taiwan Sugar Co.) and foie gras powder (#7573, NTN) in a 1:1 ratio. Adults were maintained at 28°C and 75 ± 5% relative humidity under a 12-h light/dark cycle and constantly provided a 10% sucrose solution to ensure that mosquito development was not affected at any stage.

### sgRNA design

The sgRNA of *CTL16* for CRISPR/Cas9 recognition was designed by using the web tool CRISPR Optimal Target Finder on the flyCRISPR web site (https://flycrispr.org/target-finder/) to identify the optimal CRISPR target sites and evaluate their specificity. A 599-bp genomic sequence of *CTL16* genomic sequences was got from *A. aegypti* genome (AaegL5) in VectorBase and used as the template for the CRISPR target. First, the whole template sequence of *CTL16* was pasted into the search window, and *A. aegypti* was selected as the reference genome for TagScan (53). Second, the parameter for identifying all CRISPR targets, including those with 5′-G for U6 promoter driving or 5′-GG for T7 promoter driving, was selected. All sequences with similarity to the CRISPR target queries on both strands of the G*CTL16* genomic sequence were identified by specificity, along with location information and a Genome Browse link for each potential off-target site. To generate a frameshift mutation as close as possible to the ATG site, a specific CRISPR target with zero off-target located on the antisense strand of *CTL16*, 5′- GTTGTCGCATGTCTGCTGAGAGG-3′, was selected and introduced into an *Aae*U6 promoter driving plasmid for sgRNA construction. The user manual for CRISPR Optimal Target Finder is available at https://flycrispr.org/wp-content/uploads/2019/07/flyCRISPR-Optimal-Target-Finder-Manual-29Jul14.pdf.

### pBFv-*AaeU6_CTL16*-sgRNA vector construction

A PCR-amplified 520 bp *AaeU6* promoter was generated from the *AAEL017763* locus of *A. aegypti* genomic DNA and fused with the gRNA backbone sequence to obtain an *AaeU6*-sgRNA DNA fragment by primer extension using *AeU6*-sgRNA-F1_5′-GCTTGATATCGAATTCCTATATAATTTAATTCCACTAGAGT-3′, *AeU6*-gRNA-R1_5′-TAGCTCTAAAACGGAGACGAACTCCGTCTCCATTTCACTACTCTTGCCTCTGCTCTTATA-3′, and *AeU6*-gRNA-R2_5′-TTTCAAGTTGATAACGGACTAGCCTTATTTTAACTTGCTATTTCTAGCTCTAAAACGGAG-3′. This *AeU6*-gRNA DNA fragment was cloned into the EcoRI/NotI sites of pBFv-U6.2 plasmid to create a pBFv-*AaeU6*-gRNA backbone vector for the target site (sgRNA) cloning in *A. aegypti* (54). A CRISPR/Cas9 target of the *CTL16* CDS sequence, 5′-GTTGTCGCATGTCTGCTGAGAGG-3′, was identified using CRISPR Optimal Target Finder. The *CTL16*-sgRNA fragments were generated by primer annealing using *CTL16*-sgRNA-F_5′-AAATGTTGTCGCATGTCTGCTGAG-3′ and *CTL16*-sgRNA-R_5′-AAACCTCAGCAGACATGCGACAAC-3′. All primers used in this study were synthesized by Integrated DNA Technologies. The annealed *CTL16*-sgRNA fragments were cloned into the *B*smbI sites of the pBFv-*AaeU6*-gRNA vector to generate the pBFv-*AaeU6_CTL16*-sgRNA plasmid.

### Generation of mutant mosquitoes

To establish the *CTL16*^−/−^ mutant mosquito lines, donor and helper plasmids were mixed with injection buffer (5 mM KCl and 0.1 mM NaH_2_PO_4_, pH 6.8) and then microinjected into preblastodermal-stage embryos, as described previously (31). The injected embryos were hatched and reared under standard conditions. After hatching, the surviving injection generation 0 (G0) was sexed to obtain male and female virgins. Each surviving injected G0 male adult was outcrossed with three control females, and all eggs from these control females were pooled together as a male family. G0 females were pooled together and crossed with control males at a male/female ratio of 1:3 (55), and eggs were collected from individual females as female families. G1 pupae were sequenced to identify heterozygous mutant lines, which were then reared and allowed to produce G2 offspring. These offspring were further sequenced to identify mutant lines. This process was repeated until homozygous mutant lines were established and used as the experimental groups in the subsequent studies.

### Protein extraction and western blot analysis

Protein extracts from mosquito heads, thorax, abdomen, and SGs were lysed with a mixture of radioimmunoprecipitation assay (RIPA) buffer and sample loading buffer, and then boiled for 10 min. Protein extracts were separated by sodium dodecyl sulfate–polyacrylamide gel electrophoresis (SDS–PAGE) on a 12% acrylamide gel and transferred to polyvinylidene difluoride membranes. The membranes were then blocked with 5% skim milk in Tris-buffered saline with Tween-20 (TBST) for 30 min at room temperature, washed with TBST for 10 min for thrice, and then incubated with anti-*CTL16* primary antibodies (GeneTex) overnight at 4°C. After washing three times with TBST, the membranes were incubated with 1:10,000 dilution of horseradish peroxidase-conjugated secondary antibody in 1% skim milk for 60 min. After 60 min of washing with TBST, the labeled proteins were visualized with enhanced chemiluminescence reagents.

### Quantitative real-time PCR

Total RNA was isolated using TRIzol reagent (Sigma-Aldrich) following the manufacturer's protocol. RNA quantity was evaluated using a microvolume spectrophotometer (NanoDrop ND-2000; Thermo Fisher Scientific). After DNase treatment, cDNA was reverse transcribed from 0.5 μg total RNA using SuperScript III Reverse Transcriptase (Thermo Fisher Scientific). The synthesized cDNA was amplified by PCR in 10 μL reaction mixtures using a ViiA 7 Real-Time PCR System (Applied Biosystems) with KAPA SYBR FAST qPCR Master Mix (2×) Rox low Kit (Sigma-Aldrich) for gene expression level detection. The relative mRNA expression levels were measured by the ^ΔΔ^Ct method in triplicate and normalized to ribosomal protein *S7* levels (*RPS7*; AAEL009496). The primer sequences used in the experiments are listed in Table 1.

**Table 1.** Primers used for a real-time PCR analysis of DENV-2 and innate immune pathways.

Name	Forward primer sequence	Reverse primer sequence
DENV-2	TGCACATTACCACCGCTAAGA	ATGTCCGGCTGTGACCAAG
Rps7	AGATGAACTCGGACCTGAAG	GGGACGTAGATCACGATAGCC
Dome	AAACGGTGGCAAAATGAACT	CATACAGCCGGCTTTCTTCT
Hop	GCTGGTAGTAATGCTTCGAGTGAGT	GCCGGTGCTGTAATGACTAGAA
Toll9A	TCAGTCGATGGTGCCAGTTC	CGTGGCCACTTGATGTAGGT
MyD88	GGCGAGGGTTGTTTCAAGTA	TCCCATCTGTCGATTAAGCC
Defensin	CCCGAAAGGACCAACCATGA	TTTGCAAAAGGGCGAGCTTC
FADD	GGGACCGTCGAACACTTCTT	CACTCAGCTGCATTAACCGC
Vago	CAGTAGCATTTGCCGGTCAGA	CGATGTTGGATCGTAGCACTTC
Ago2	ACAACAGCAACAATCCCAGA	GTGGACGTTGATCTTGTTGG

### Plaque formation assay

Viral plaque formation assays for both virus stocks and infected mosquitoes were performed according to previously published protocols (56). To determine the virus titer in SGs, 2 × 10^5^ BHK cells were seeded into a six-well plate and incubated overnight. SG from each mosquito was ground and diluted with serum-free Dulbecco's modified Eagle medium (DMEM). Two hours after infection, the unbound viral particles were removed and overlaid with 3 mL DMEM containing 1% methylcellulose and 2% fetal bovine serum (FBS, Gibco). After 6 days of incubation, cells were fixed and stained with 0.5 µL cell staining solution (0.5% crystal violet, 1.85% formaldehyde, 50% ethanol, and 0.85% NaCl) and then washed with water. The number of plaques was counted, and the virus titer in the SGs of each mosquito was determined as plaque-forming units (PFUs) in the SGs of each mosquito.

### AGB6 mice rearing and mosquito-to-mouse virus transmission

All animal experiments were performed using AGB6 mice (types I and II deficient in interferon signaling) lacking both interferon-α/β receptor and interferon-γ receptor (C57BL/6 mice background). AGB6 mice were provided by Dr Guann-Yi Yu's lab (NHRI, Taiwan) and consisted of male mice 8–10 weeks of age (57, 58). The animal protocol was approved by the Institutional Animal Care and Use Committee of the National Health Research Institutes (NHRI-IACUC-107054-A). All mice were bred and maintained at the ABSL-2 animal feeding room in the Laboratory Animal Center of the NHRI; the Center received full accreditation from the Association for Assessment and Accreditation of Laboratory Animal Care (AAALAC) International in 2015. The mice were then shaved on the ventral side and put in a mosquito-housing cage for mosquito feeding. Each mouse was bitten by four mosquitoes. Mouse serum was collected every 2 dpi to test for infectious virus particles and to conduct serum cytokine expression assays. Mice were monitored daily for survival, weight loss, and disease symptoms.

### Immunofluorescence assay

To determine *CTL16* and DENV E-protein expression, mosquitoes were collected on days 5–14 following thoracic injection. Wild-type and mutant mosquitoes were collected and dissected in 1× phosphate-buffered saline (PBS) to obtain SGs, then fixed with 4% paraformaldehyde in PBS for 30 min at room temperature, washed once with 1× PBS, rinsed for 20 min three times in PBST (0.3% Triton-X in PBS), and incubated in 0.1% PBST overnight at 4°C. SGs were then blocked in 2% bovine serum albumin (2% bovine serum albumin in 0.1% PBST) for 30 min at room temperature and incubated with primary antibodies overnight at 4°C. After washing with 0.1% PBST, the samples were incubated with secondary antibody (Alexa Fluor 647 conjugated secondary antibody) diluted in 0.1% PBST for 1 h at room temperature, then washed again with 0.1% PBST and incubated with dye (DAPI, phalloidin, and wheat germ agglutinin) diluted in 0.1% PBST for 1 h at room temperature. After incubation with secondary antibodies, SGs were rinsed and washed three times with 0.1% PBST. The samples were mounted in VectaShield Antifade Mounting Medium for analysis, and images were captured with a Leica TCS SP5 II confocal microscope.

### Harvesting the infected mosquito saliva and transmission efficiency assay

Saliva was collected on 7 dpi following thoracic infection. The wings and legs of mosquitoes were removed to prevent their escape, and the mosquito mouthpiece was inserted into a P200 tip filled with 5 µL FBS. After 30 min, the FBS in the tip was added to 45 µL serum-free medium in Roswell Park Memorial Institute (RPMI), and plaque formation assay was performed. Mosquito tissues were also subjected to plaque formation assay. Transmission efficiency was calculated as follows (59):


$$\begin{array}{rl} & \mathrm{Transmission}\;\mathrm{efficiency}\;=\\ & \;\frac{\mathrm{Number}\;\;\mathrm{of}\;\mathrm{mosquitoes}\;\;\mathrm{with}\;\mathrm{positive}\;\;\mathrm{infectious}\;\;\mathrm{saliva}}{\mathrm{Number}\;\;\mathrm{of}\;\mathrm{examined}\;\;\mathrm{mosquito}} \end{array}$$

### Plasmid construction

The *Ae*Pub promoters were amplified from pMOS1-*Ae*Pub-DENV all in one-8miR-3xp3-eGFP plasmid using the PCR primers pAc5.1_3xHA fusion *Ae*Pub-pr_F and pAc5.1_3xHA fusion *Ae*Pub-pr_R. Agarose gel-purified PCR products of *Ae*Pub promoter fragments were subcloned into BglII/SphI double-digested pAc5.1 vector with C-terminal fused 3xHA tag using an In-Fusion HD Cloning Kit (Clontech) to obtain the p*Ae*Pub-3xHA transition vector. The AAEL000533 (*CTL16*) CDS PCR fragments without stop codon were generated by PCR of the cDNA pools from *A. aegypti* Liverpool strain using the primers pPub_3xHA fusion AAEL000533-CDS_F and pPub_3xHA fusion AAEL000533-CDS_R (Table 2). The agarose gel-purified AAEL000533-CDS fragments were cloned into the SphI/XhoI sites of pAc5.1 vector with C-terminal-fused 3xHA tag using the In-Fusion HD Cloning Kit to generate the p*Ae*Pub-AAEL000533-3xHA plasmid.

**Table 2.** List of pPub-CTL16-3xHA vector screening primers.

Primer name	Sequence
pAc5.1_3xHA fusion *Ae*Pub-pr_F	TCATTTTTCCAGATCTTATCTTTACATGTAGCTTGTGCATTGAATC
pAc5.1_3xHA fusion *Ae*Pub-pr_R	GGTGCGTTGAGCATGCGTTGAAATCTCTGTTGAGCAGAAAAAGAAA
pPub_3xHA fusion AAEL000533-CDS_F	GAGATTTCAACGCATCCTAGGATGGCTCTTTCATTATATCTAATC
pPub_3xHA fusion AAEL000533-CDS_R	CGTATGGGTACTCGAGCGCCTGTTCGCACACAAAGCGACGCATCG

### C6/36 cell transfection and DENV-2 infection

First, 3 × 10^6^ C6/36 cells were seeded into six-well plates and incubated overnight. Then, diluted plasmid was added to 9 µL of Lipofectamine 2000 reagent diluted in Opti-MEM medium (1:1 ratio) and incubated for 20 min at room temperature. Next, the plasmid–lipid complex was added to the cells and incubated for 4 h at 28°C, then washed once with 1× PBS and replaced with DMEM containing 10% FBS, followed by incubation for 2 days. The transfected C6/36 cells were infected with DENV-2 at a multiplicity of infection of 0.1 for 2 h, then washed once with 1× PBS and replaced with DMEM containing 10% FBS, followed by incubation for 2 days, and these cells were harvested for western blotting to check viral protein accumulation.

### Immunoprecipitation assay

To determine the binding ability of *CTL16* to DENV-2, 3 μg of recombinant *CTL16* protein (self-manufacture) or GFP protein (Abcam) was pre-incubated with DENV (5 × 10^6^ PFU) in a reaction volume of 500 μL RIPA buffer at 4°C for 1 h. Subsequently, the protein–virus mixture was incubated with 2 μL anti-*CTL16*, and a negative control was conducted with 3 μL anti-GFP antibody overnight at 4°C. This was followed by immunoprecipitation with 20 μL protein A/G magnetic beads (Thermo Fisher Scientific) for 3 h at 4°C. The samples were then boiled and resolved by 15% SDS–PAGE. Primary antibodies, including anti-E (4G2) (1:2,000), anti-NS1 (1:2,000), anti-*CTL16* (1:1,000), or anti-GFP (1:2,000), were utilized for the western blot analysis.

### Statistical analysis

All animals in the experiment were randomized, and each experiment was performed at least three times, infecting at least five AGB6 mice per experiment. Normally distributed datasets were evaluated using two-way ANOVA, while non-normally distributed datasets were evaluated using a Mann–Whitney test (GraphPad Prism version 8.0). Confocal images were processed using ImageJ. The mean fluorescence intensity of E protein in the SG was quantified, and ImageJ was used to measure the identified area and calculate total fluorescence intensity (The Open Lab Book, https://theolb.readthedocs.io/en/latest/index.html) using the following formula:


$$\begin{array}{rl} & \mathrm{Corrected}\;\mathrm{total}\;\mathrm{cell}\;\mathrm{fluorescence}\;=\\ & \;\mathrm{Integrated}\;\mathrm{density}\text{–}(\mathrm{Area}\;[\mathrm{distal}\;\mathrm{lateral}\;\mathrm{lobes},\\ & \;\mathrm{proximal}\;\mathrm{lateral}\;\mathrm{lobes},\\ & \;\mathrm{and}\;\mathrm{medial}\;\mathrm{lobes},\mathrm{respectively}]\;\mathrm{of}\;\mathrm{selected}\;\mathrm{cell}\\ & \;\times \;\mathrm{mean}\;\mathrm{fluorescence}\;\mathrm{of}\;\mathrm{background}\;\mathrm{readings}) \end{array}$$

## Supplementary Material

pgae188_Supplementary_Data

## Data Availability

All relevant data are provided in the manuscript and its Supplementary material.
