# Supplementary material for: Effect of C-type lectin 16 on dengue virus infection in Aedes aegypti salivary glands
Source: PNAS Nexus. 2024 May 16;3(5):pgae188. doi: 10.1093/pnasnexus/pgae188 (PMC11134184; doi:10.1093/pnasnexus/pgae188)
Supplement: pgae188_Supplementary_Data [file pgae188_supplementary_data.pdf]

Supplementary data

Figure S1. Salivary gland morphology under a dissecting microscope. (A) Comparison of control and *CTL16*$^{-/-}$ mutant (right) salivary glands (n=5), scale bar = 250 μm. **(B)** Salivary gland area (mm^2) calculated using ImageJ. Data are represented as mean ± SD. ns: no significant difference.

(A)

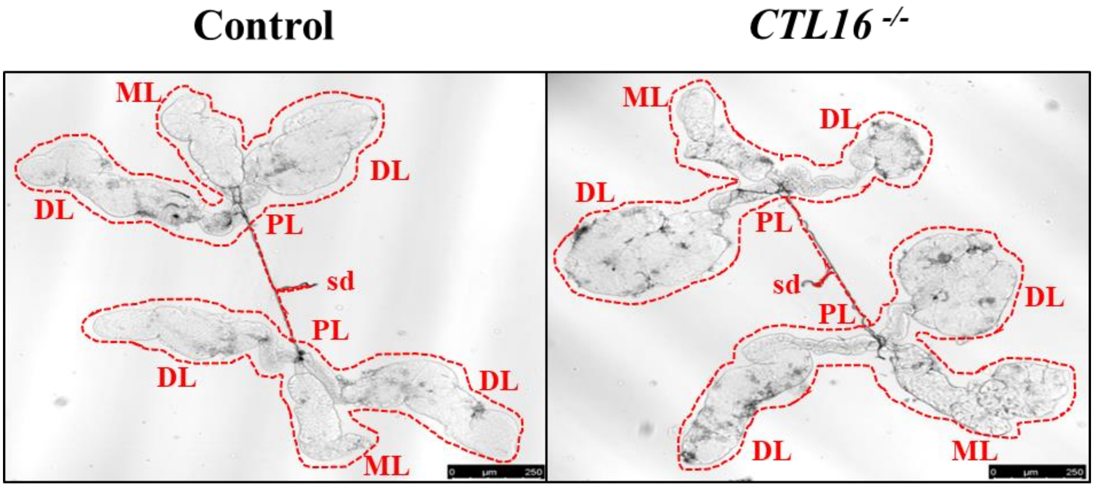

(B)

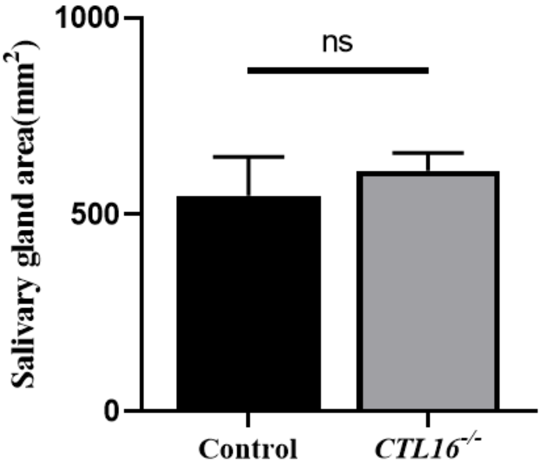

Figure S2. DENV challenge experiment in *CTL16*$^{-/-}$ mutant mosquitoes. DENV-2 challenge experiment in *CTL16*$^{-/-}$ mutants at 7 days post-infection (dpi). **(A)** The salivary glands and **(B)** the saliva was collected from female mutant and wild type mosquitoes. In each experiment, fifteen mosquitoes were checked with three replicate tests conducted. Viral titer was determined from plaque assays using BHK21 cells. The viral titer for each mosquito is plotted, and the standard error of the mean is indicated. A Mann–Whitney rank sum test was used to analyze the difference in virus titers. **(C)** The transmission efficiencies of the *CTL16*$^{-/-}$ mosquitoes are indicated as percentages and defined as the number of positive saliva samples (i.e., successful transmission events) divided by the number of tested samples. A *t*-test was performed to analyze potential differences in the mosquito transmission efficiency assay. Asterisks represent significant differences between the genotypes: *p <0.05, **p <0.01, ***p <0.001, ****p <0.0001.

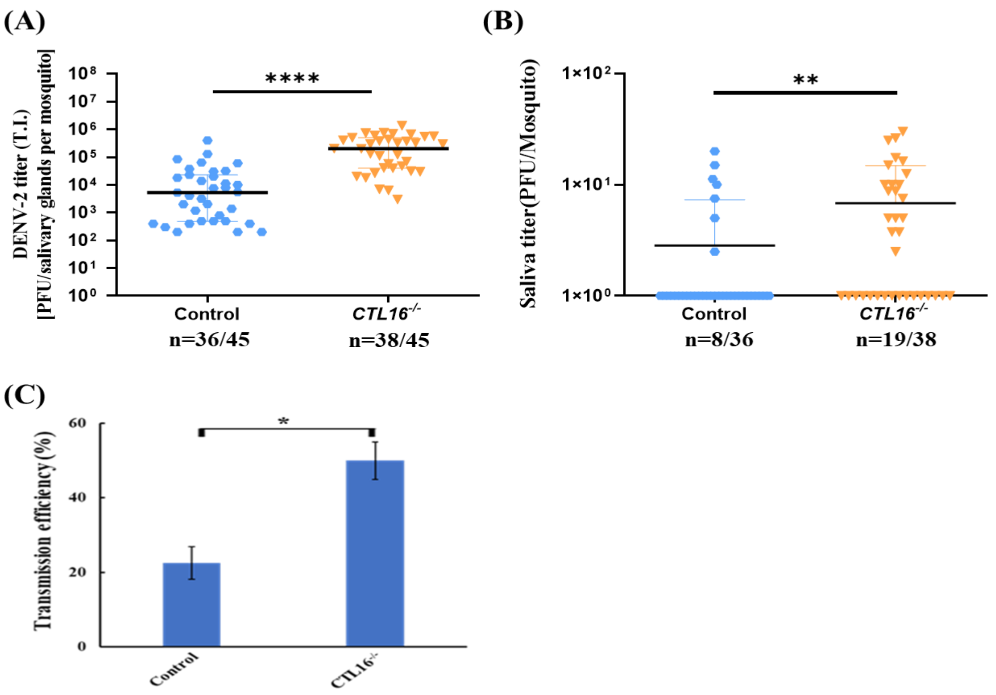

Figure S3. DENV E protein expression level in distal-lateral lobes of salivary glands from *CTL16*$^{-/-}$ mosquitos post-thoracic injection. **(A)** 10 days post-injection (dpi) and **(B)** 14 dpi; on the right is the quantitative analysis of immunostaining. DENV E protein expression in control (AAEL006511-Cas9 founder line) and *CTL16*$^{-/-}$ female salivary glands. After thoracic injection, salivary glands (n=40) were collected and subjected to immunofluorescence staining. Scale bar = 250 μm. Asterisks represent significant differences between the genotypes (Mann-Whitney test; *p <0.05). ns: no significant difference, DL: distal-lateral lobes, PL: proximal-lateral lobes, ML: medial lobes.

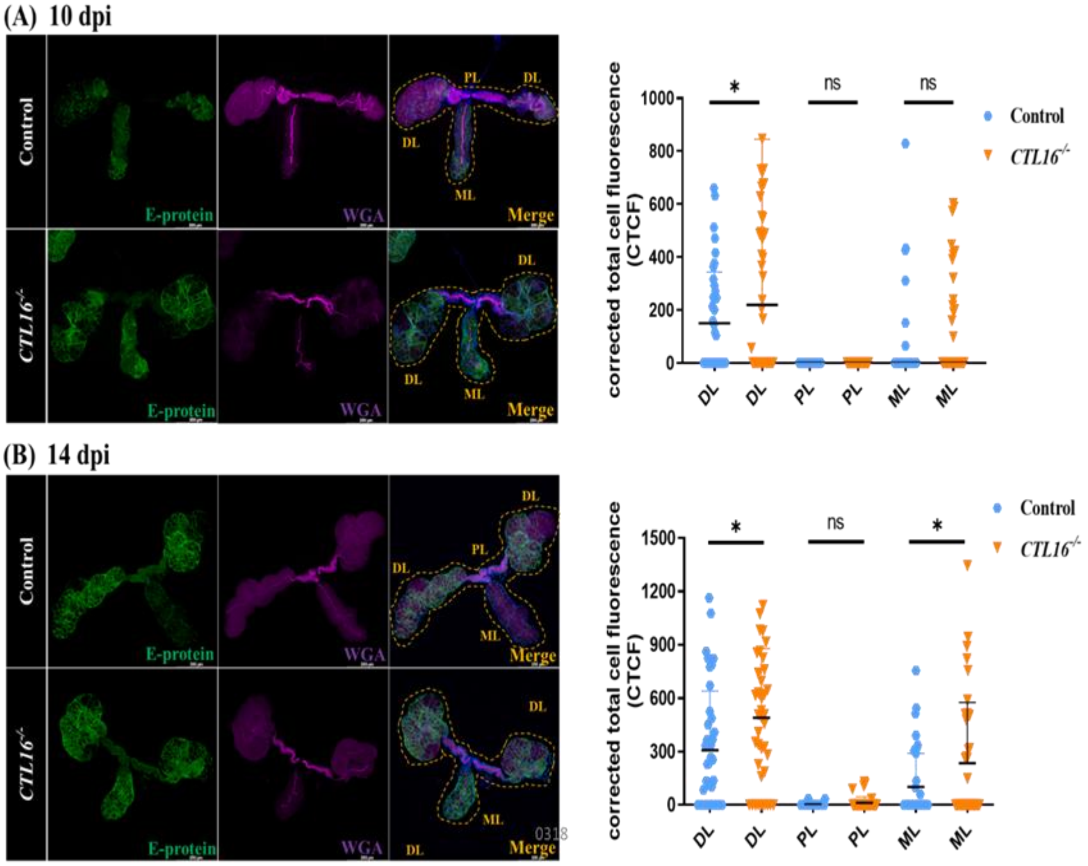

Figure S4. Activation of immune pathways was relatively lower in the *CTL16*$^{-/-}$ mutants after thoracic challenge with DENV2. (A) Salivary glands (SGs) from pre-injection (D0) and 1-, 2-, 5-, and 7-days post-infection (dpi) mosquitoes were analyzed to determine the relative expression of immune pathway components by qPCR, using RPS7 as the reference gene. We examined the immune response in the whole three distinct lobes of the salivary gland: for the separated response from each lobe, (B) the distal lateral lobe, (C) the proximal lateral lobe, and (D) the medial lobe. Each time point sample corresponds to three replicates (5 mosquitoes for each group). Expression levels of Dome and Hop (JAK/STAT pathway), FADD and Vago (IMD pathway), Toll9A, MyD88, and Defensin (Toll immune pathway), and Ago2 (RNAi pathway factors) are shown. Comparisons were performed using *t*-tests. Asterisks represent significant differences: *p<0.05, **p<0.01. Control: AAEL006511-Cas9 founder line.

(A)

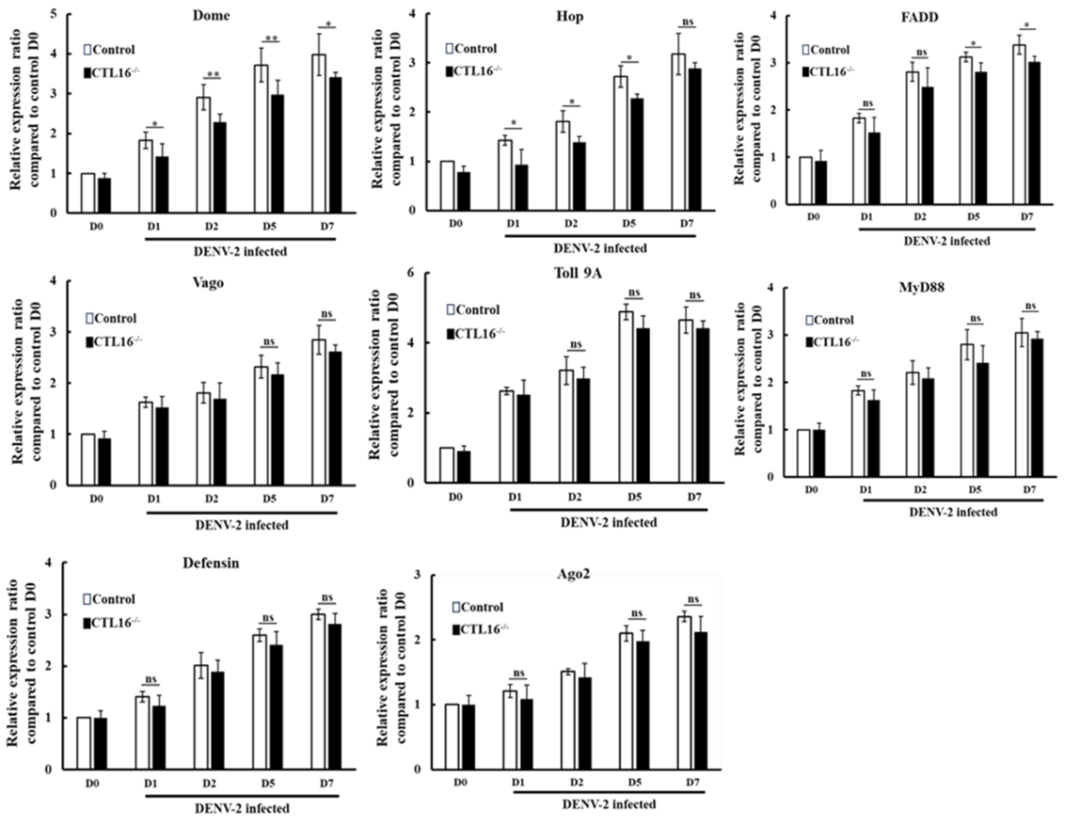

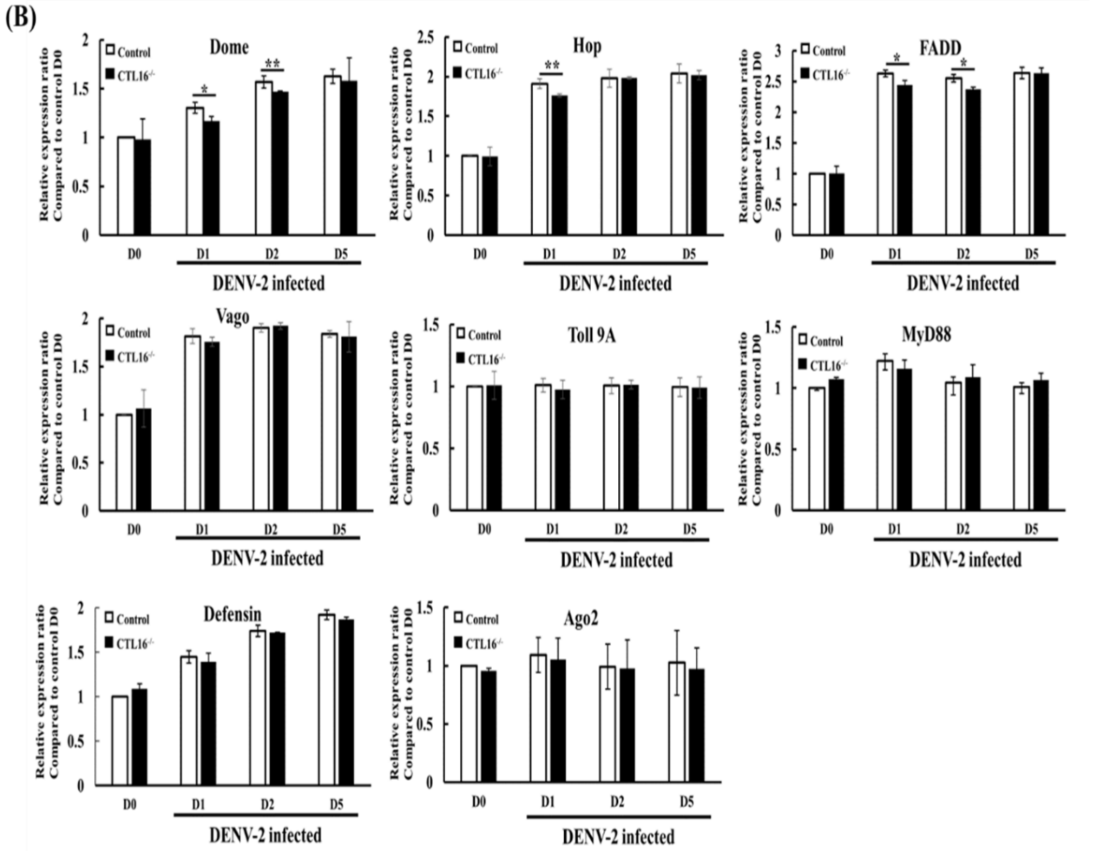
(B)
Dome
Hop
FADD
Vago
Toll 9A
MyD88
Defensin
Ago2
Control
CTL16-/-
Relative expression ratio Compared to control D0
D0
D1
D2
D5
DENV-2 infected
*
**

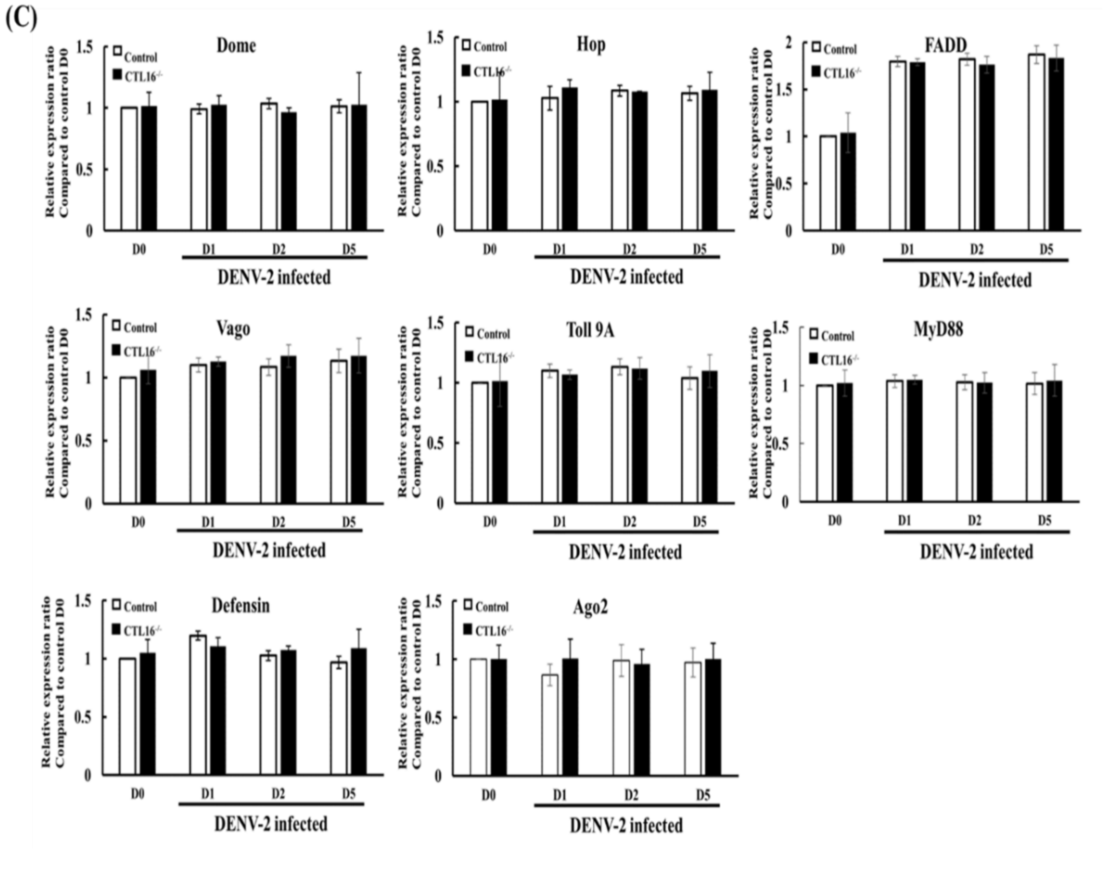
(C)
Dome
Hop
FADD
Vago
Toll 9A
MyD88
Defensin
Ago2
Control
CTL16-/-
Relative expression ratio Compared to control D0
D0
D1
D2
D5
DENV-2 infected

(D)

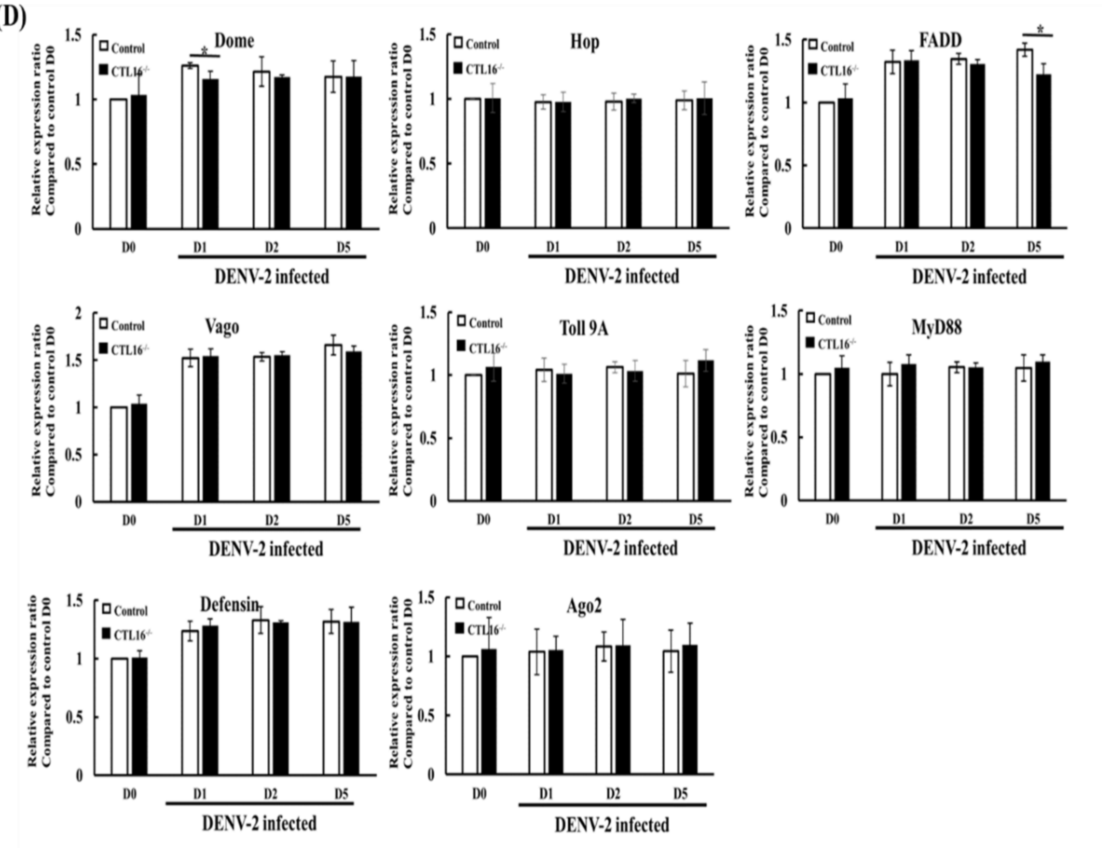
Dome
Hop
FADD
Vago
Toll 9A
MyD88
Defensin
Ago2
Control
CTL16-/-
Relative expression ratio
Compared to control D0
D0
D1
D2
D5
DENV-2 infected
